# Supplementary material for: An automated 13.5 hour system for scalable diagnosis and acute management guidance for genetic diseases
Source: Nat Commun. 2022 Jul 26;13:4057. doi: 10.1038/s41467-022-31446-6 (PMC9325884; doi:10.1038/s41467-022-31446-6)
Supplement: Supplementary file 1 — Supplemenary Information [file 41467_2022_31446_MOESM1_ESM.pdf]

Supplementary Information File

An automated 13.5 hour system for scalable diagnosis and acute management guidance for genetic diseases

Mallory J. Owen, Sebastien Lefebvre, Christian Hansen, Chris M. Kunard, David P. Dimmock, Laurie D. Smith, Gunter Scharer, Rebecca Mardach, Mary J. Willis, Annette Feigenbaum, Anna-Kaisa Niemi, Yan Ding, Luca Van Der Kraan, Katarzyna Ellsworth, Lucia Guidugli, Bryan R. Lajoie, Timothy K. McPhail, Shyamal S. Mehtalia, Kevin K. Chau, Yong H. Kwon, Zhanyang Zhu, Sergey Batalov, Shimul Chowdhury, Seema Rego, James Perry, Mark Speziale, Mark Nespeca, Meredith S. Wright, Martin G. Reese, Francisco M. De La Vega, Joe Azure, Erwin Frise, Charlene Son Rigby, Sandy White, Charlotte A. Hobbs, Sheldon Gilmer, Gail Knight, Albert Oriol, Jerica Lenberg, Shareef A. Nahas, Kate Perofsky, Kyu Kim, Jeanne Carroll, Nicole G. Coufal, Erica Sanford, Kristen Wigby, Jacqueline Weir, Vicki S. Thomson, Louise Fraser, Seka S. Lazare, Yoon H. Shin, Haiying Grunenwald, Richard Lee, David Jones, Duke Tran, Andrew Gross, Patrick Daigle, Anne Case, Marisa Lue, James A. Richardson, John Reynders, Thomas Defay, Kevin P. Hall, Narayanan Veeraraghavan, Stephen F. Kingsmore.

Supplementary Tables

Table S1. Precision and recall of phenotypic features extracted by CNLP from EHRs in 10 children with genetic diseases.
Table S2. Characteristics of 4 retrospective cases used to test performance of the 13.5 hour automated sequencing and interpretation pipeline.
Table S3. Analytic performance of 3 automated interpretation software systems, MOON (InVitae), GEM (Fabric Genomics) and Trusight Software Suite (Illumina), in 4 retrospective cases and one prospective case.
Table S4. Concordance and interventions reviewed, retained, and deleted for 15 GTRx pilot genes that underwent independent review by five expert biochemical geneticists.
Table S5. Fields used in the curation of gene-condition associations.
Table S6. Results from a survey of nine clinicians on the user interface of GTRx.

Supplementary Data

Supplementary Data 1. 563 genetic disorders associated with 357 genes identified as presenting in the pediatric population and having available treatment. Abbreviations: NBS, Newborn Screening. ORPHA., Orphanet. DEF., deficiency or deficient. HERED., hereditary. SUSC., susceptibility. CONG., congenital. AR, autosomal recessive. AD, autosomal dominant. FAM., familial. SYN., syndrome. INF., infancy. −, negative. W/W, with or without. GEN., generalized. NEO., neonatal. PROG., progressive. XL, X-linked. &, and. ASSOC W., associated with. SEV., severe. AUT., autosomal. D.T., due to. COMP., complementation group. COMB., combined. ABN., abnormalities or anomalies. +, POSITIVE. DIS., disease, or disorder.
Supplemental Data 2. Variables included in the RedCap instance used to review interventions pulled by automatic and manual curation.
Supplemental Data 3. The 8,889 interventions curated, reviewed, and retained or rejected in 563 genetic disorders. Concordance value N/A signifies no secondary review performed for this set of interventions.

Supplementary Figures

Figure S1: Screenshot from Genome-To-Treatment (GTRx) for Timothy Syndrome-*CACNA1C*
Figure S2: Screenshot of Acute Treatment Detail from Genome-To-Treatment (GTRx) for Timothy Syndrome-*CACNA1C*

Figure S3. Screenshot from GTRx for Hereditary Fructose Intolerance-*ALDOB*
Figure S4. Genome-To-Treatment (GTRx) RedCap review system for Timothy Syndrome-*CACNA1C*. Select Interventions For Review page.
Figure S5. Genome-To-Treatment (GTRx) RedCap review system for Timothy Syndrome-*CACNA1C*. Intervention / Treatment page for Propanolol.
Figure S6. Genome-To-Treatment (GTRx) RedCap review system for Timothy Syndrome-*CACNA1C*. List Of Disease Treatment Publications page.
Figure S7. Genome-To-Treatment (GTRx) RedCap review system for Timothy Syndrome-*CACNA1C*. Secondary Review page.

Supplementary Methods
GTRx General Principles for Intervention Review and Adjudication

Table S1. Precision and recall of phenotypic features extracted by NLP from EHRs in 10 children with genetic diseases. Precision=tp/tp+fp. Recall=tp/tp+fn. Abbreviations: EIEE: Early Infantile Epileptic Encephalopathy; AD: Autosomal Dominant; AR: Autosomal Recessive; DN: de novo; P: Pathogenic; LP: Likely Pathogenic; S: Singleton; T: Trio; I: Inherited; U: undetermined; OMIM: Online Mendelian Inheritance in Man; CF: Clinical Feature; Inh: Inheritance; n.a.: not applicable; WES: whole exome sequencing.

Family	S or T	WES or WGS	Disease	Affected Gene	MIM ID	Inh	DN or I	Variant 1 (V1)	Variant 2 (V2)	V1 P/ LP	V2 P/ LP	DOL NLP extract	Sex	Consan-guinity	NLP Features	NLP Precision	NLP Recall
201	T	WES	Prader Willi Syndrome	15q11-q13 del	176270	AD	DN	Chr15:23684685-26108259del	n.a.	P		4	♀	U	89	0.53	0.95
205	T	WGS	Dursun Syndrome	*G6CP3*	612541	AR	I	c.207dupC, p.Ile70Hisfs	c.199_218+1delCTCAA CCTCATCTTCAAGTGG	P	P	2	♂	No	94	0.93	0.95
213	S	WGS	Visceral Heterotaxy 5	*NODAL*	270100	AD	I	c.778G>A, p.Gly260Arg	n.a.	LP		3	♂	U	89	0.90	0.98
233	T	WGS	Tuberous Sclerosis 1	*TSC1*	191100	AD	DN	c.1498C>T, p.Arg500Ter	n.a.	P		5	♀	No	167	0.57	0.95
243	T	WGS	Pyridoxine dependent seizures	*ALDH7A1*	266100	AR	I	c.328C>T, p.Arg110Ter	c.1279G>C, p.Glu427Gln	P	P	6	♂	No	36	0.97	0.50
6094	T	WGS	Argininosuccinic aciduria	*ASL*	207900	AR	I	c.706C>T, p.Arg236Trp	c.706C>T, p.Arg236Trp	P	P	4	♀	Yes	55	0.85	0.87
6098	T	WGS	Gaucher disease	*GBA*	230800	AR	I	c.1503C>G, p.Asn501Lys	c.1448T>C , p.Leu483Pro	LP	P	215	♀	No	112	0.92	0.94
6108	T	WGS	Tuberous Sclerosis 2	*TSC2*	613254	AD	DN	c.935_936delTC, p.Leu312Glnfs	n.a.	P		4	♂	No	86	0.76	0.98
7003	T	WGS	EIEE6	*SCN1A*	607208	AD	DN	c.5555T>C, p.Met1852Thr	n.a.	P		424	♂	U	67	0.81	0.93
7004	T	WGS	Hypertrophic cardiomyopathy 1	*MYH7*	192600	AD	I	c.746G>A, p.Arg249Gln	n.a.	P		5171	♂	U	99	0.68	0.96
**Average**															**89.4**	**0.79**	**0.90**
Standard Deviation															35.3	0.15	0.14

Table S2. Characteristics of four retrospective and three prospective cases used to test performance of the 13.5-hour automated sequencing and interpretation pipeline (Table 1, Figure 4). Abbreviations: R: Retrospective; Pr: Prospective; AD: Autosomal Dominant; DN: de novo; P: Pathogenic; LP: Likely Pathogenic; M: Male; F: Female; S: Singleton; T: Trio; Inh: Inherited; XL: X linked; Het: Heterozygous; Hom: Homozygous; Hem: Hemizygous; OMIM: Online Mendelian Inheritance in Man; n.a.: not applicable; n.k.: not known; MT: mitochondrial; HP: heteroplasmic; D: Duo.

Analysis	Subject ID	S or T	Disease	Gene	OMIM ID	Inheri -tance	Zygosity	*de novo* or inher -ited	Variant 1 (V1)	Variant 2 (V2)	V1 P/ LP	V2 P/ LP	Age at enrollment (days)	Sex	Parents Consan- guinous
Retro- spective	AG928	S	Hereditary Fructose Intolerance	*ALDOB*	229600	AR	Het	n.k	c.448G>C, p.Ala150Pro	c.524C>A, p.Ala175Asp	P	P	107	M	N
	AG366	S	Ornithine Transcarbamylase Defiency	*OTC*	311250	XL	Hem	*De Novo*	c.275G>A, p.Arg92Gln	n.a.	P	na	5	M	N
	AF414	S	Propionic Acidemia	*PCCA*	606054	AR	Hom	n.k	c.1899+4_1899+7del	n.a.	LP	na	4	F	N
	AI003	T	Developmental and epileptic encephalopathy 11	*SCN2A*	613721	AD	Het	*De Novo*	c.4437G>C, p.Gln1479His	n.a.	LP	na	7	F	N
Pro- spective	AH638	T	Thiamine metabolism dysfunction syndrome 2	*SLC19A3*	607483	AR	Hom	Inh	c.597dup, H200fs	n.a.	P	na	42	M	Y
	CSD59F	D	Leigh syndrome	*MT-ATP6*	256000	MT	HP	*De Novo*	m.8993T>C, p.Leu156Pro	n.a.	P	na	6	M	N
	CSD709	S	Geleophysic dysplasia	*ADAMTSL2*	231050	AR	Het	Inh	c.338G>T, p.Arg113Leu	c.1851C>A, p.Cys617Ter	P	LP	1	M	N

Table S3. Analytic performance of 3 automated interpretation software systems (MOON (InVitae), GEM (Fabric Genomics) and Trusight Software Suite (Illumina)) in 4 retrospective cases and one prospective case. *Includes processing time for DRAGEN v3.7. Abbreviations: SNV: single nucleotide variant; SV: structural variant; CNV: copy number variant.

Case Number	AG928	AI115	AI148	AI185	AH638	Average
Run	1020	1204	1208	1218	1026	
Type of case		Retrospective			Prospective	
Diagnosis	*ALDOB*	*OTC*	*PCCA*	*SCN2A*	*SLC19A3*	
**MOON (InVitae)**						
Rank of correct diagnosis	1	1	1	1	1	1
SNVs	6	7	9	4	10	7.2
SV/CNVs	20	1	11	8	0	8
Total	26	8	20	12	11	15.4
Time to provisional diagnosis (min)	9	10	12	10	10	10.2
**GEM (Fabric Genomics)**						
Rank of correct diagnosis	3	1	1	4	1	2
Ranked variants (including SV/CNVs)	5	6	5	16	8	8
Time to provisional diagnosis (min)	39	43	44	40	48	42.8
**Trusight Software Suite (Illumina)**						
Rank of correct diagnosis	1	1	1	1	1	1
SNVs	5	2	2	5	15	5.8
SV/CNVs	0	0	0	0	0	0
Total	5	2	2	5	15	5.8
Time to provisional diagnosis (min)*	213	230	178	276	220	223.4

Table S4. Concordance and interventions reviewed, retained and deleted in the 15 GTRx pilot genes that underwent independent review by 5 expert biochemical geneticists.

Condition	OMIM ID	Gene Affected	Total Interventions Curated	Interventions with concordant reviews	Concordance	Interventions retained after adjudication	Original interventions retained	Interventions deleted	Interventions deleted
Timothy Syndrome	601005	*CACNA1C*	19	14	74%	8	42%	11	58%
Primary aldosteronism, seizures, & neurologic abnormalities	615474	*CACNA1D*	6	4	67%	5	83%	1	17%
Carnitine Acylcarnitine Translocase Deficiency	212138	*SLC25A20*	8	6	75%	8	100%	0	0%
Maple Syrup Urine Disease	248600	*DBT*	14	4	29%	9	64%	5	36%
Congenital Myasthenic Syndrome	614750	*DPAGT1*	12	2	17%	1	8%	11	92%
Glycogen Storage Disease III	232400	*AGL*	5	1	20%	3	60%	2	40%
Tyrosinemia Type 1	276700	*FAH*	6	4	67%	3	50%	3	50%
Fructose 1,6 Bisphosphatase Deficiency	229700	*FBP1*	6	4	67%	4	67%	2	33%
Glycogen Storage Disease 1a	232200	*G6PC*	21	7	33%	6	29%	15	71%
Glycogen Storage Disease Ib	232220	*SLC37A4*	15	4	27%	5	33%	10	67%
Glycogen Storage Disease II	232300	*GAA*	24	17	71%	7	29%	17	71%
Galactosemia II	230200	*GALK1*	1	1	100%	1	100%	0	0%
Hereditary Fructose Intolerance	229600	*ALDOB*	2	1	50%	1	50%	1	50%
Ornithine Transcarbamylase Deficiency	311250	*OTC*	22	10	45%	13	59%	9	41%
Propionic Acidemia	606054	*PCCA*	29	15	52%	14	48%	15	52%
**Average**			**12.7**	**6.3**	**52.8%**	**5.9**	**54.9%**	**6.8**	**45.1%**
**Standard Deviation**			**8.7**	**5.3**	**24.1%**	**4.1**	**25.9%**	**6.0**	**25.9%**

Table S5. Fields used in the curation of gene-condition associations.

Responsible entity	Field	Description	Guidance Provided
Automated data pull	HGNC gene id		
Automated data pull	HGNC gene symbol		
Automated data pull	condition_id	OMIM ID if no OMIM use Orphanet ID. OMIM:<ID> or ORPHA:<ID>	
Automated data pull	condition_name	OMIM name if no OMIM use Orphanet name	
Curator	intervention_excerpt	abbreviation of excerpt or excerpt from the evidence source that mentions the acute case intervention	Focus on pediatric acute cases and if little evidence expand to human acute cases then to human cases. No animal models.
Curator	intervention_type	possible values = medicine, surgery, device, diet, other	
Curator	drug_name	molecule, drug, formulation	
Curator	drugbank_id	DrugBank ID	
Curator	intervention_evidence_PMID	list of PubMed ids	supporting evidence of the intervention for the given gene-condition
Curator	category_of_evidence	list of value from (1a, 1b, 2a, 2b, 2c, 3a, 3b, 3c, 4, 5)	Category of Evidence supporting clinical utility of intervention from www.ncbi.nlm.nih.gov/pubmed/10037645
Curator	effectiveness_of_intervention	C = Curative, E = Effective, or A = Amerliorative	

Table S6. Results of nine clinician surveys of perceptions of GTRx.

SURVEY QUESTION (10 POINT LIKERT SCALE, 1=LOWEST, 10=HIGHEST)	MEDIAN RESPONSE	MINIMUM	MAXIMUM
Q1. WOULD YOU USE THIS WEBSITE AS A CLINICIAN?	9	3	10
Q2. HOW EASY IS THE WEBSITE TO USE?	9	8	10
Q3. HOW EASY IS IT TO FIND THE INFORMATION YOU'D LIKE TO SEE?	9	5	10
Q4. HOW USEFUL IS THE INFORMATION PROVIDED?	6	5	10
**SURVEY QUESTION (5 POINT LIKERT SCALE, 1=LOWEST, 5=HIGHEST)**	**Median Response**	**Minimum**	**Maximum**
Q5. OVERALL, HOW WELL DOES GTRX MEET YOUR NEEDS?	3 (Somewhat)	2 (Not so well)	4 (Very)
Q6. HOW VISUALLY APPEALING IS GTRX?	4 (Very)	3 (Somewhat)	5 (Extremely)
Q7. HOW EASY IS IT TO UNDERSTAND THE INFORMATION ON THE WEBSITE?	4 (Very)	3 (Somewhat)	5 (Extremely)

Supplementary Figures

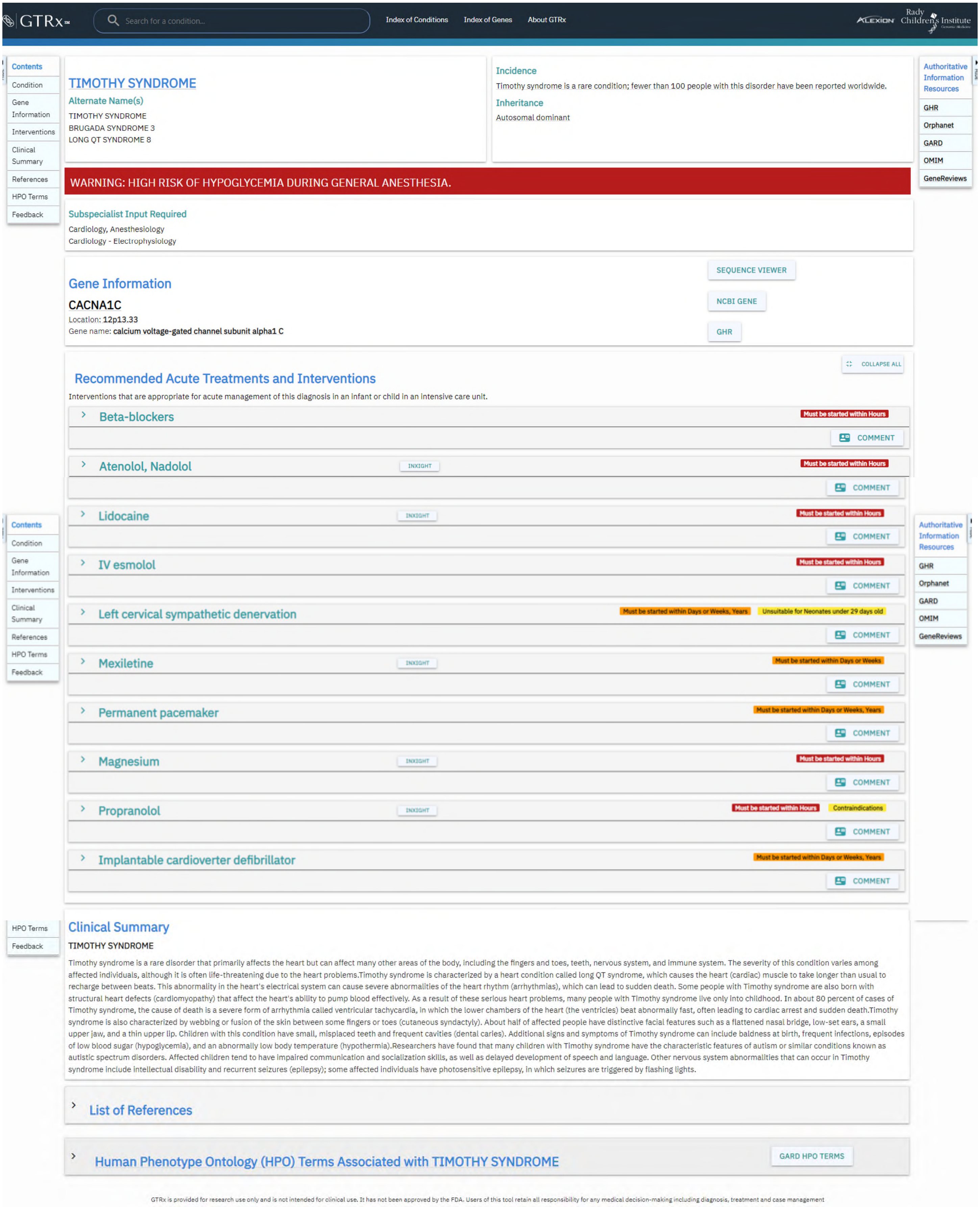

Figure S1: Screenshot from Genome-To-Treatment (GTRx) for Timothy Syndrome-*CACNA1C*

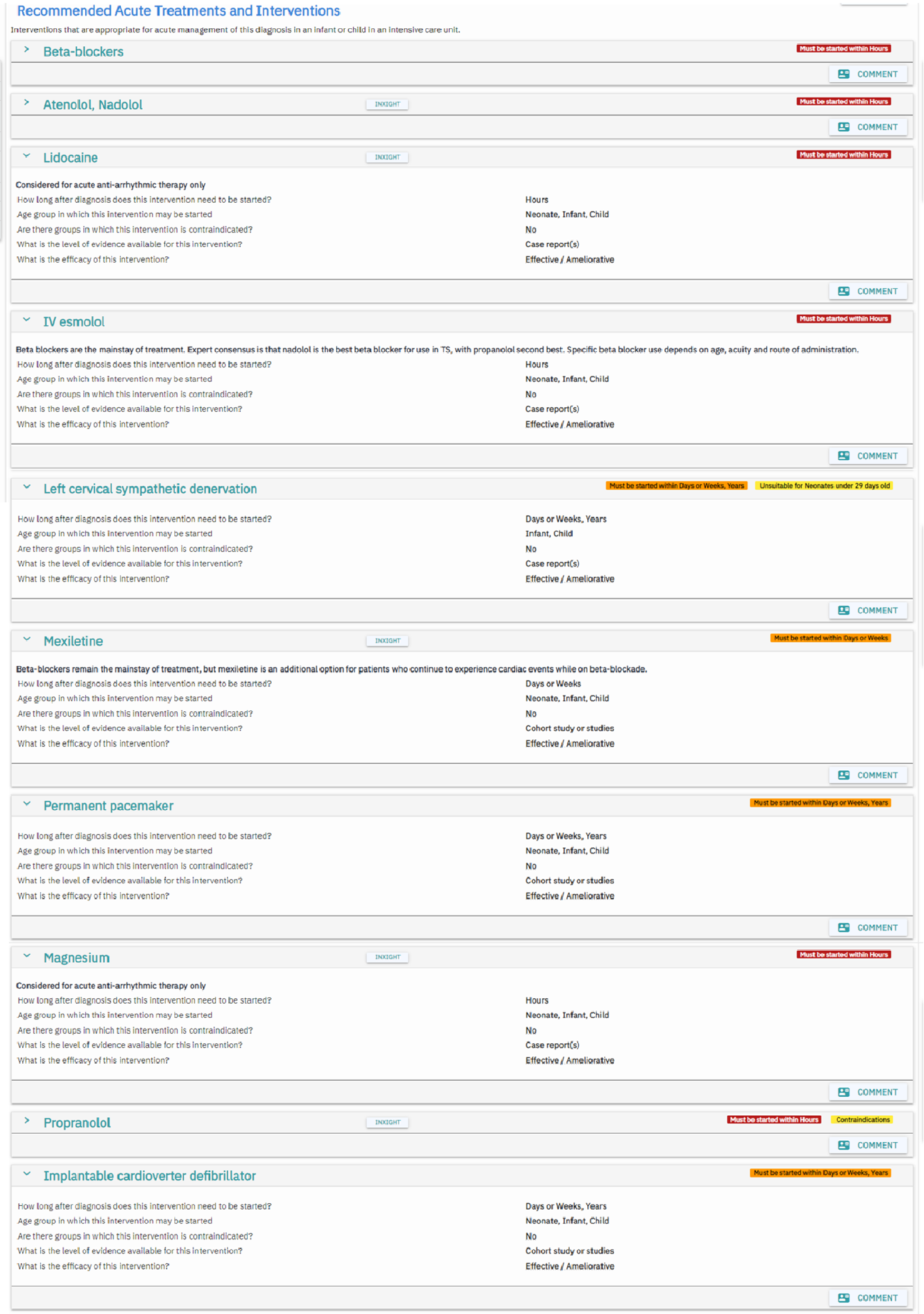

Figure S2: Screenshot of Acute Treatment Detail from Genome-To-Treatment (GTRx) for Timothy Syndrome-*CACNA1C*

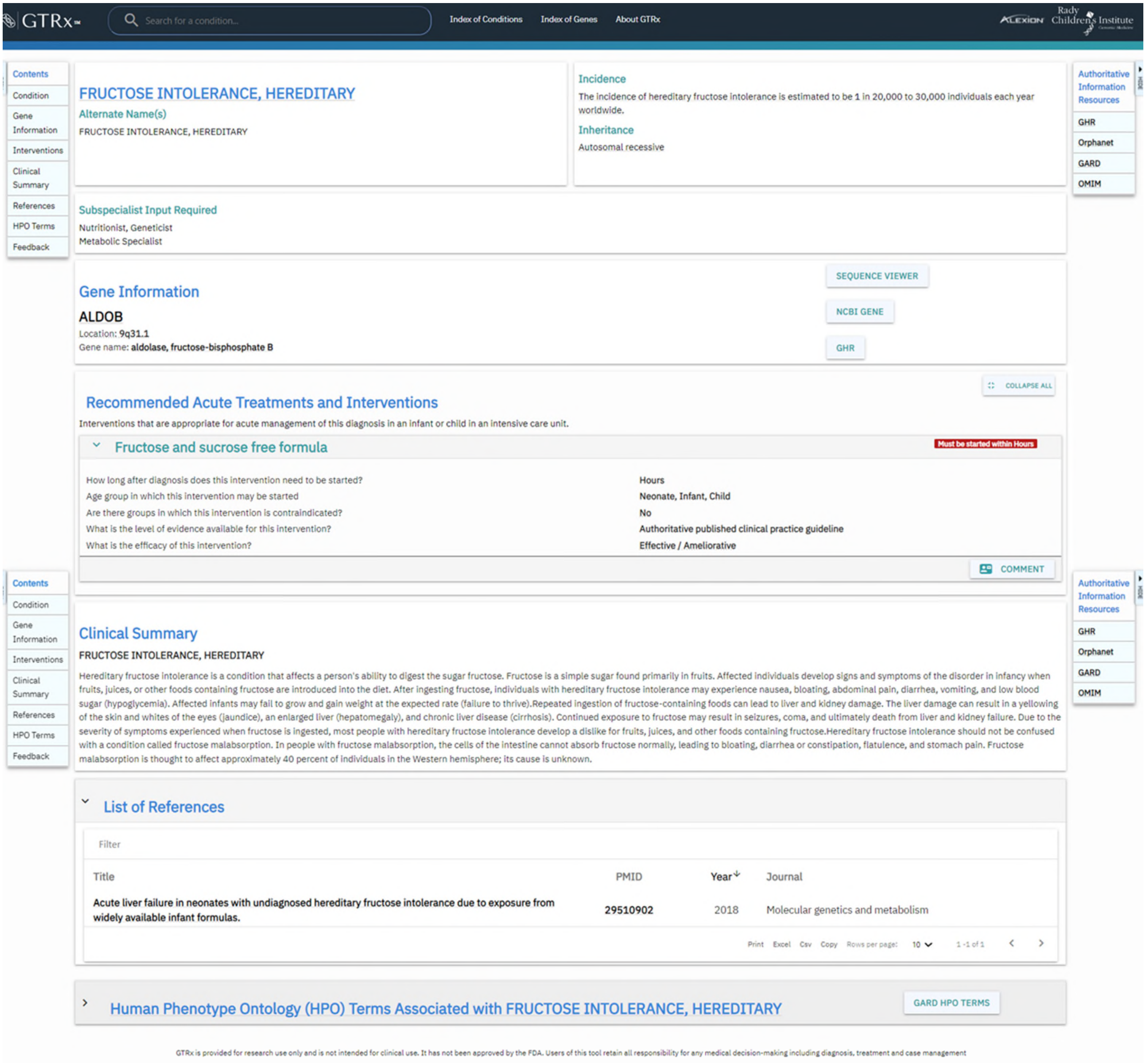

Figure S3. Screenshot from GTRx for Hereditary Fructose Intolerance-*ALDOB*

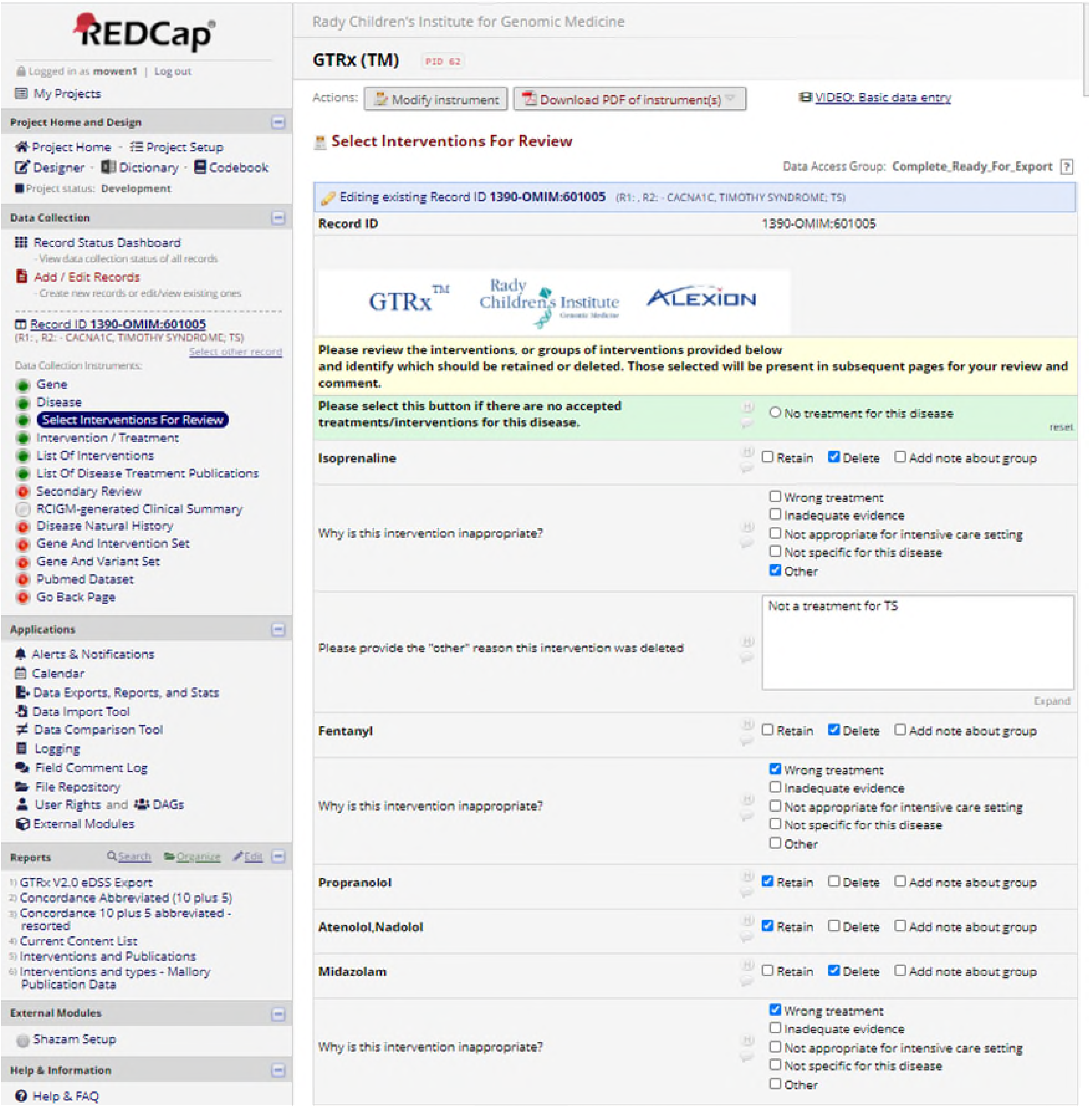

Figure S4. Genome-To-Treatment (GTRx) RedCap review system for Timothy Syndrome-*CACNA1C*. Select Interventions For Review page.

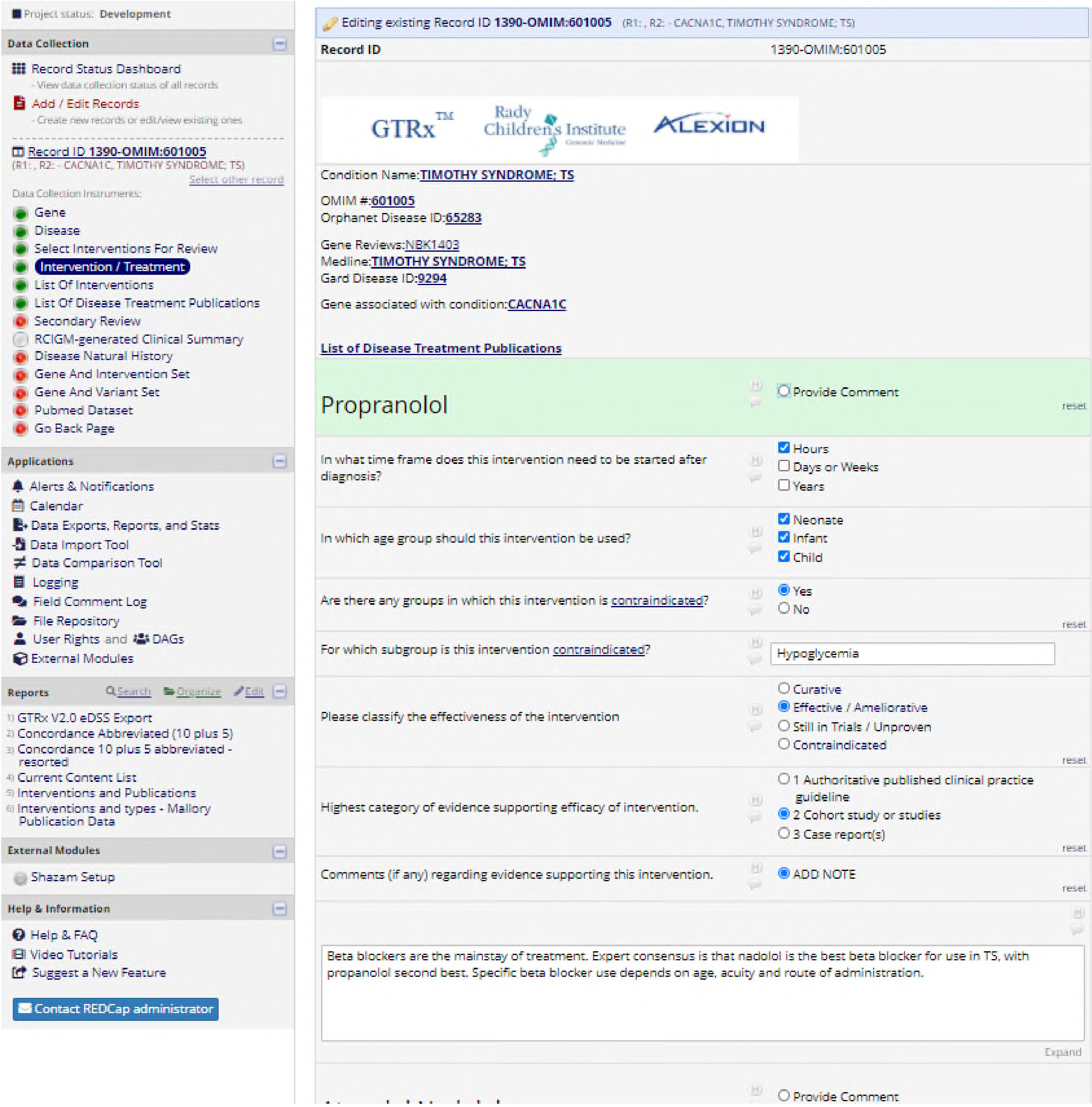

Figure S5. Genome-To-Treatment (GTRx) RedCap review system for Timothy Syndrome-*CACNA1C*. Intervention / Treatment page for Propanolol.

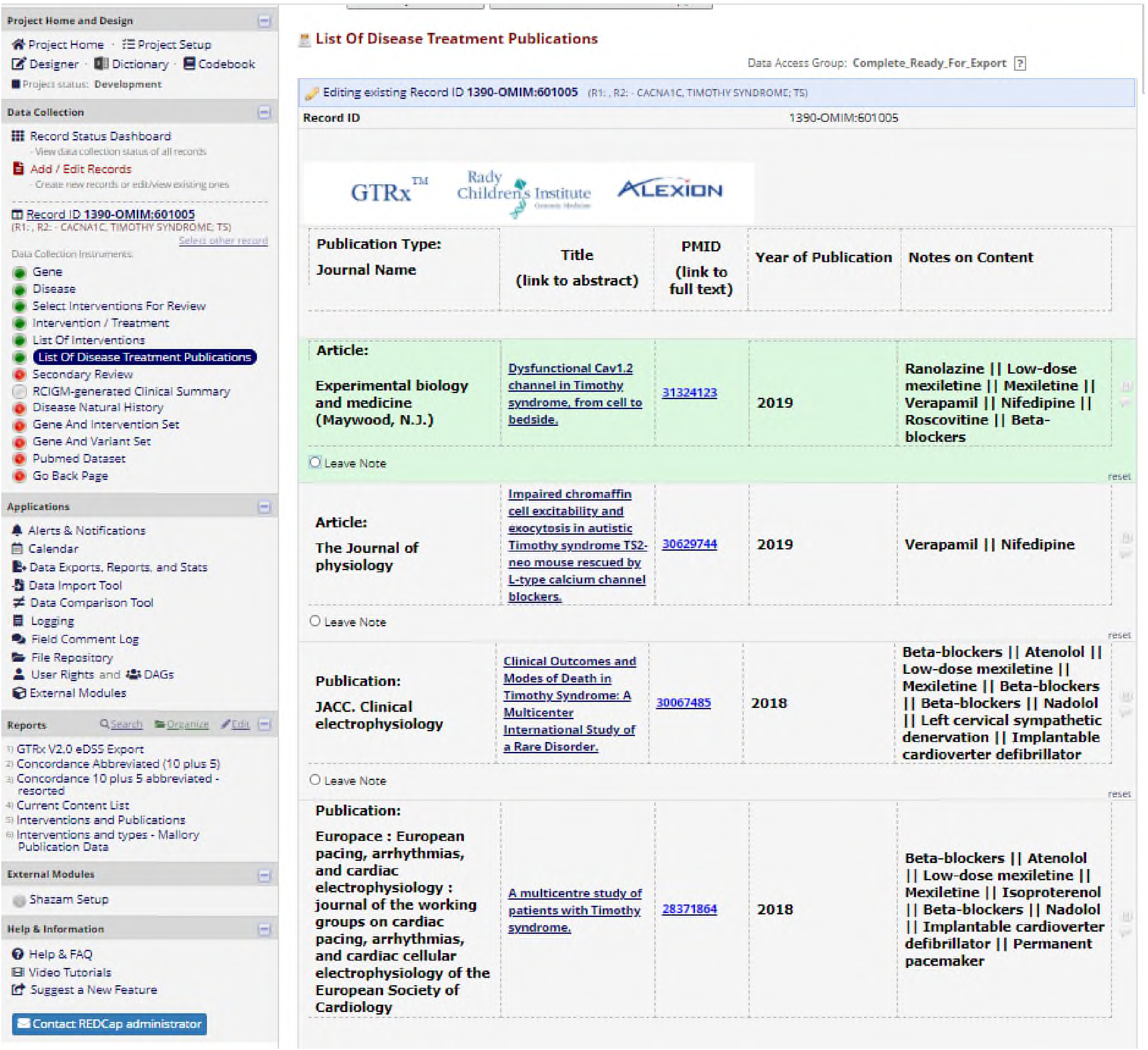

Figure S6. Genome-To-Treatment (GTRx) RedCap review system for Timothy Syndrome-*CACNA1C*. List Of Disease Treatment Publications page.

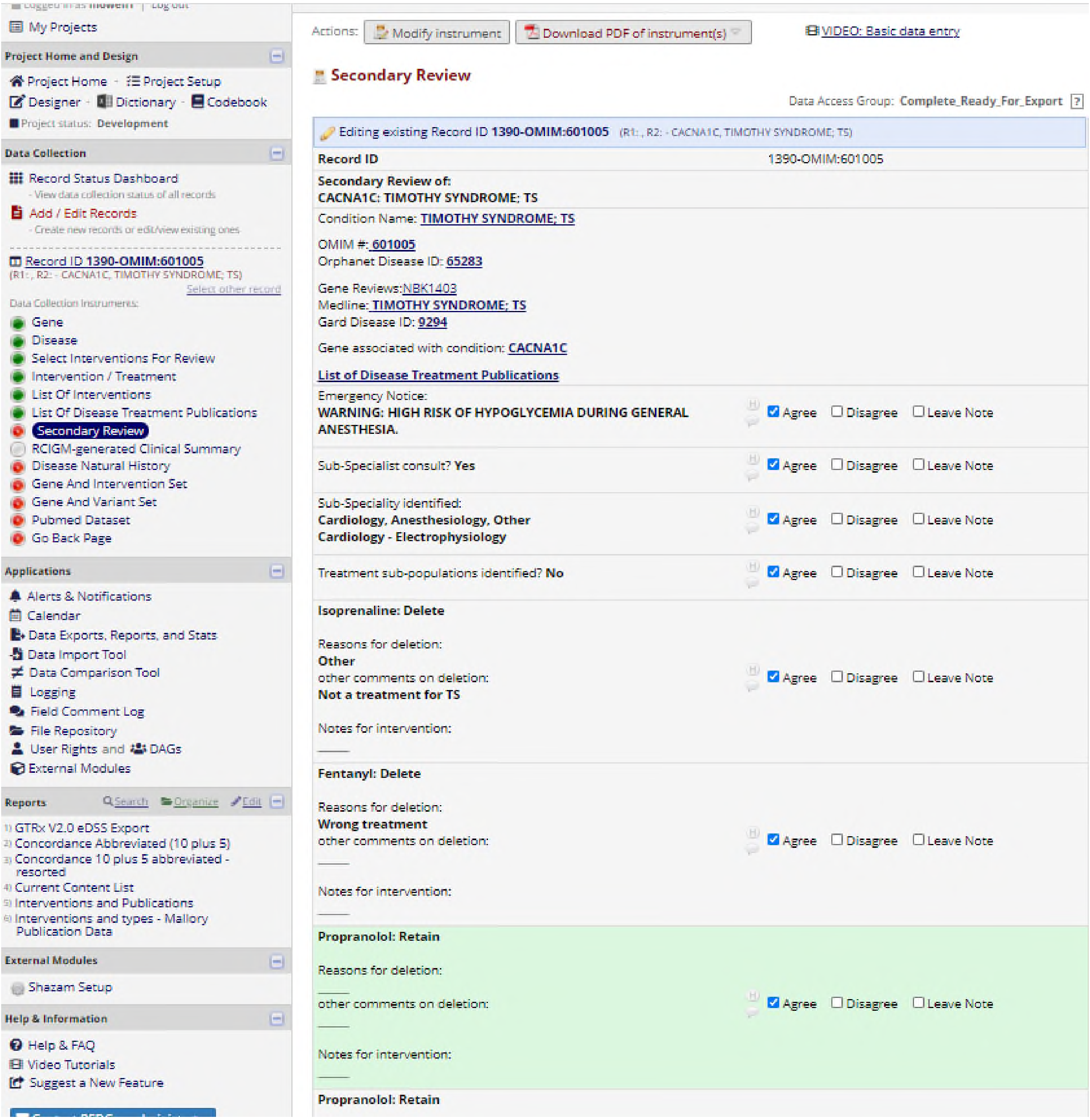

Figure S7. Genome-To-Treatment (GTRx) RedCap review system for Timothy Syndrome-*CACNA1C*. Secondary Review page.

Supplementary Methods

GTRx General Principles for Intervention Review and Adjudication

1. Our scope is focused on providing **information for intensivists in an acute care setting** facing a new diagnosis of a rare genetic condition. Interventions that fall outside of this scope will not appear in the interventions list on the GTRx tool.
2. Our intent is **to include all interventions for which published evidence of efficacy or ameliorative effect in the specific disease being considered** is available.
    a. Interventions that are not the first line, but for which evidence does exist, **will be** included.
3. Our scope includes treatments that are **used for condition-specific supportive care**, but not for general supportive care as would be routinely performed in an ICU setting.
    a. Any intervention in which this distinction is unclear will be discussed and a consensus reached by the expert panel before inclusion.
4. Interventions that fall outside of the scope set out in 1 will be collected in a separate database, which will be periodically re-reviewed as new evidence becomes available. At such time when sufficient evidence accumulates to support the inclusion of these interventions, they will be added to the GTRx interface. Evidence will be re-reviewed on an ongoing basis each year.
