# Supplementary material for: An automated 13.5 hour system for scalable diagnosis and acute management guidance for genetic diseases
Source: Nat Commun. 2022 Jul 26;13:4057. doi: 10.1038/s41467-022-31446-6 (PMC9325884; doi:10.1038/s41467-022-31446-6)
Supplement: Supplementary file 6 — Reporting Summary [file 41467_2022_31446_MOESM6_ESM.pdf]

nature research

Corresponding author(s): Stephen F. Kingsmore

Last updated by author(s): November 22,2021, May 25, 2022

Reporting Summary

Nature Research wishes to improve the reproducibility of the work that we publish. This form provides structure for consistency and transparency in reporting. For further information on Nature Research policies, see our Editorial Policies and the Editorial Policy Checklist.

Statistics

For all statistical analyses, confirm that the following items are present in the figure legend, table legend, main text, or Methods section.

n/a	Confirmed	
☐	☒	The exact sample size (*n*) for each experimental group/condition, given as a discrete number and unit of measurement
☐	☒	A statement on whether measurements were taken from distinct samples or whether the same sample was measured repeatedly
☒	☐	The statistical test(s) used AND whether they are one- or two-sided<br>*Only common tests should be described solely by name; describe more complex techniques in the Methods section.*
☐	☒	A description of all covariates tested
☐	☒	A description of any assumptions or corrections, such as tests of normality and adjustment for multiple comparisons
☐	☒	A full description of the statistical parameters including central tendency (e.g. means) or other basic estimates (e.g. regression coefficient) AND variation (e.g. standard deviation) or associated estimates of uncertainty (e.g. confidence intervals)
☐	☒	For null hypothesis testing, the test statistic (e.g. *F*, *t*, *r*) with confidence intervals, effect sizes, degrees of freedom and *P* value noted<br>*Give P values as exact values whenever suitable.*
☒	☐	For Bayesian analysis, information on the choice of priors and Markov chain Monte Carlo settings
☒	☐	For hierarchical and complex designs, identification of the appropriate level for tests and full reporting of outcomes
☒	☐	Estimates of effect sizes (e.g. Cohen's *d*, Pearson's *r*), indicating how they were calculated

Our web collection on statistics for biologists contains articles on many of the points above.

Software and code

Policy information about availability of computer code

Data collection	Electronic health record data was extracted with CliX ENRICH v.6.7 (CliniThink Inc.) natural language processing software. Genome sequencing was performed NovaSeq 6000 instruments featuring custom software to accelerate cycle time (Illumina). Genome sequence alignment and nucleotide variant detection used DRAGEN software (v.3.7.1, Illumina). Sample, sample preparation and sequencing metadata was collected with a custom Laboratory Information Management system (L7 Informatics). We obtained datasets from Online Mendelian Inheritance in Man (OMIM, https://www.omim.org/downloads), Orphanet v5.47 (http://www.orphadata.org/cgi-bin/img/PDF/OrphadatOnRequestProductsDescription.pdf), Genetic and Rare Disease Information Center (GARD, https://rarediseases.info.nih.gov/), GeneReviews (https://ftp.ncbi.nlm.nih.gov/pub/litarch/ca/84/), Genetics Home Reference (GHR, now MedLinePlus Genetics, https://medlineplus.gov/about/developers/geneticsdatafilesapi/), DrugBank v5.0 (https://go.drugbank.com/releases/5-0-1), MedGen (https://ftp.ncbi.nlm.nih.gov/pub/medgen/), Medscape (https://emedicine.medscape.com/), NORD's Rare Disease Database (https://rarediseases.org/for-patients-and-families/information-resources/rare-disease-information/), ClinVar (https://ftp.ncbi.nlm.nih.gov/pub/clinvar/), ClinicalTrials.gov (https://clinicaltrials.gov/AllPublicXML.zip), the Cochrane Database of Systematic Reviews (https://www.cochranelibrary.com/help/access), and PubMed. Transformation pipelines for these datasets were created with the Konstanz Information Miner (KNIME v4.3 https://docs.knime.com/). We developed a web resource, Genome-To-Treatment (GTRx, http://gtrx.rbsapp.net/) to automatically display this information and link it to automated WGS results on a gene-by-gene basis.
Data analysis	Genome sequence alignment and nucleotide variant detection used DRAGEN software (v.2.5.1, Illumina). Sequence data was transferred between analysis steps an automated pipeline (Axolotl v.5.0, Rady Children's Institute for Genomic Medicine). Automated variant interpretation was performed in parallel using MOON v3.3.4 (InVitae), GEM v1 (Fabric Genomics), and the Illumina TruSight Software Suite v2.0 (Illumina). Annotation sources and versions were ClinVar: 2021-03-02, dbNSFP: 4.0, dbSNP: 151, dbscSNV: 1.1, Apollo: 2021-03-08, RefSeq: 37, gnomAD: 2.1.1, HPO: 2021-02-08, KB: 2021-05-07, DGV: 2016-03-01, dbVar: 2019-07-07, Mitomap: 2019-01-14, Mitimpact: 2.9.1, Mastermind: 2021-01-02, InvitaeKB: 2021-02-25, HGMD Professional 2020. Precision, recall and F1 score of structural variants and copy number variants used Witty.er v0.3.4 (Illumina, https://github.com/Illumina/witty.er/blob/master/docs/release-notes/README.md). Variant benchmarking was with NIST gold standard variant sets for SNVs and indels (NISTv4.1), and SVs and CNVs (NISTv0.6, https://github.com/

jzook/genome-data-integration/tree/master/StructuralVariants/NISTv0.6.). For each genetic disease selected for inclusion in Genome-to-Treatment, we indexed the full text of all MEDLINE/PubMed references that mentioned a drug, device, diet or surgery used to treat the disease using three artificial-intelligence based search engines (Mastermind, Genomenon; Rancho Biosciences, Epam Systems). Resultant, manually curated datasets and links to the information resource were integrated into a custom Research Electronic Data Capture (REDCap, Vanderbilt University, https://redcap.radygenomiclab.com/redcap_v10.6.3/ProjectSetup/index.php?pid=62) survey for expert review. Following review, the retained interventions and qualifying statements were incorporated into the GTRx information resource (http://gtrx.rbsapp.net).

For manuscripts utilizing custom algorithms or software that are central to the research but not yet described in published literature, software must be made available to editors and reviewers. We strongly encourage code deposition in a community repository (e.g. GitHub). See the Nature Research guidelines for submitting code & software for further information.

Data

Policy information about availability of data

All manuscripts must include a data availability statement. This statement should provide the following information, where applicable:

- Accession codes, unique identifiers, or web links for publicly available datasets
- A list of figures that have associated raw data
- A description of any restrictions on data availability

All data associated with this study are present in the paper, Supplementary Materials, or are available at the Longitudinal Pediatric Data Resource under a material transfer agreement or data use agreement, as appropriate, and subject to the limitations of the informed consent documents for each subject (Accession Number nbs000003.v1.p, https://www.nbstrn.org/research-tools/longitudinal-pediatric-data-resource). The GTRx interface is available as a research use only version here http://gtrx.rbsapp.net/. InterVar is available at Github (https://github.com/WGLab/InterVar). CLIXEnrich is available from CliniThink (info@clinithink.com). Moon is available from Diploid (info@diploid.com). The DRAGEN Platform and the Illumina TruSight Software Suite are available from Illumina (Shyamal Mehtalia, smehtalia@illumina.com, www.illumina.com). OPAL and GEMS are available from Fabric Genomics (info@fabricgenomics.com). The RCIGM portal, Axolotl pipeline and REDCap instance are available from Christian Hansen (chansen@rchsd.org). The KNIME pipeline is available from Sebastien Lefebvre (sebastien.lefebvre@alexion.com).

Field-specific reporting

Please select the one below that is the best fit for your research. If you are not sure, read the appropriate sections before making your selection.

[x] Life sciences [ ] Behavioural & social sciences [ ] Ecological, evolutionary & environmental sciences

For a reference copy of the document with all sections, see nature.com/documents/nr-reporting-summary-flat.pdf

Life sciences study design

All studies must disclose on these points even when the disclosure is negative.

Sample size	The implementation science study design was adaptive and iterative. We have previously published increasingly rapid and automated diagnostic whole genome sequencing methods in 2012, 2016 and 2019 (referenced in the manuscript). These studies informed sample size to demonstrate analytic performance and diagnostic performance of these new methods. We also determined sample size in accordance with typical validation experiments for new steps or methods in diagnostic tests developed according to the Clinical Laboratory Improvement Amendments Act (CLIA) and guidelines of the American College of Medical Genetics (AMCG), College of American Pathologists (CAP) and the States of New York and California.
Data exclusions	We have established diagnostic quality standards for acceptance of whole genome sequencing runs, lanes, reads, variants and other metadata in accordance with the Clinical Laboratory Improvement Amendments Act (CLIA) and guidelines of the American College of Medical Genetics (AMCG), College of American Pathologists (CAP) and the States of New York and California. These standards guided reporting of data herein. For example, runs, lanes, and sequence reads that failed to pass quality metrics were excluded. We have reported the proportion of reads passing those filters (% Reads with Quality Score >30, Error rate (%), % Reads Mapped, % Duplicate Reads) in Table 1. The reported data represent all of the final results from methods at the end of 18 months of development. We have not reported data from the methods development phase.
Replication	All sampleswere tested at least once by both the novel methods & standard, CLIA-accredited diagnostic methods. Where availa ble, we used National Institute of Standards & Technology reference samples & "gold standard" reference data to compare performance of novel methods with standard methods. All replications were successful.
Randomization	Randomization was not appropriate for this quality improvement study. All samples were tested both the the novel methods, described herein, and standard, previously published methods, and results were compared.
Blinding	Blinding was not appropriate for this quality improvement study. For retrospective and prospective case analysis we wished to determine whether automated methods recapitulated manually ascertained diagnoses. The operator (laboratory director) needed to review both data sets. For retrospective cases, comparison of novel methods always followed manual, standard methods. For prospective cases, comparison of standard methods always followed novel methods.

Reporting for specific materials, systems and methods

We require information from authors about some types of materials, experimental systems and methods used in many studies. Here, indicate whether each material, system or method listed is relevant to your study. If you are not sure if a list item applies to your research, read the appropriate section before selecting a response.

Materials & experimental systems

n/a	Involved in the study
x	Antibodies
x	Eukaryotic cell lines
x	Palaeontology and archaeology
x	Animals and other organisms
	x Human research participants
	x Clinical data
x	Dual use research of concern

Methods

n/a	Involved in the study
x	ChIP-seq
x	Flow cytometry
x	MRI-based neuroimaging

Human research participants

Policy information about studies involving human research participants

Population characteristics	Indications for rapid diagnostic genome sequencing were age 0-18 years, male or female, any race/ethnicity, who were critically ill, receiving care in regional intensive care units, and with diseases of unknown etiology, and in whom a single locus genetic disease with an effective treatment was on the differential diagnosis. The 4 retrospective blood samples were from such patients, and had received standard rapid genome sequencing previously that had diagnosed a genetic disease that we sought to recapitulate with faster turnaround time using the novel methods, and 3 prospective patients, who received standard tests and novel methods in parallel. This st udy was performed as a quality improvement and received an IRB waiver as human subjects research.
Recruitment	Retrospective samples were selected on the basis of availability of remaining blood samples from newborns, infants and children (male or female, of any race or ethnicity), who were critically ill and receiving care in regional intensive care units, who had diseases of unknown etiology, had received a diagnosis of a single locus genetic disease with an effective treatment, by standard, diagnostic whole genome sequencing methods. Prospective samples were selected on the basis of being from newborns, infants and children (male or female, of any race or ethnicity), who were critically ill and receiving care in regional intensive care units, who had diseases of unknown etiology, and in whom there was the likelihood of having a single locus genetic disease with an effective treatment and in whom a delay in instituting that treatment was likely to result in additional morbidity or risk of mortality. Prospective samples also had to be of sufficient volume to permit standard diagnostic testing and the prototypic methods.
Ethics oversight	Testing of reference samples, retrospective blood samples and prospective blood samples was performed as a quality improvement project in accordance with standards for laboratory developed tests established by the Clinical Laboratory Improvement Amendments Act (CLIA) and guidelines of the American College of Medical Genetics (AMCG), College of American Pathologists (CAP) and the States of New York and California. Testing was performed under the oversight of the patient safety and regulatory compliance bodies of Rady Children's Hospital, San Diego. These methods had previously been reviewed by the Food and Drug Administration in a presubmission enquiry for an investigational device exemption and had been determined to be non-significant risk. In particular, in each of the prospective cases, the medical director of Rady Children's Institute for Genomic Medicine, made a considered determination that the benefits of accelerated diagnosis of a treatable, rapidly progressive genetic disease outweighed the risk of potential patient privacy. Standard, diagnostic, CLIA/ CAP-compliant, ultra-rapid whole genome sequencing was performed as quickly as possible in parallel. Questionnaires were administered to physicians related to the Genome-to-Treatment web resource under a research protocol approved by the Institutional Review Board of Rady Children's Hospital / University of California - San Diego.

Note that full information on the approval of the study protocol must also be provided in the manuscript.

Clinical data

Policy information about clinical studies

All manuscripts should comply with the ICMJE guidelines for publication of clinical research and a completed CONSORT checklist must be included with all submissions.

Clinical trial registration	This manuscript describes development of methods for diagnosis and acute management of children with suspected single locus genetic diseases for which effective treatments were available. The clinical data and clinical study component of this manuscript was performed as an implementation science, quality improvement study. It involved retrospective comparison of diagnostic results obtained with standard established methods (diagnostic whole genome sequencing as a CLIA/CAP compliant laboratory developed test) with novel, prototypic methods by retesting four blood samples. Secondly, it involved comparison of diagnostic results obtained with standard established methods (diagnostic whole genome sequencing as a CLIA/CAP compliant laboratory developed test) with novel, prototypic methods by parallel testing of three blood samples. The ICMJE guidelines with regard to clinical trial registration and CONSORT checklists relate to clinical research and not to quality improvement studies. The manuscript conforms to SQUIRE 2.0 guidelines (SQUIRE-EDU (Standards for QUality Improvement Reporting Excellence in Education): Publication Guidelines for Educational Improvement. Ogrinc G, Armstrong GE, Dolansky MA, Singh MK, Davies L. Acad Med. 2019 Oct;94(10):1461-1470).
Study protocol	The manuscript contains all of the details of the adaptive, iterative methods development and their prototypic use in an implementation science, quality improvement study.
Data collection	Clinical data was collected at Rady Children's Institute for Genomic Medicine and Rady Children's Hospital, San Diego between

December 2019 and June 2021.

Outcomes	The quality improvement endpoints were 1. faster time to diagnosis of single locus genetic diseases; 2. faster time to implementation of treatment of single locus genetic diseases; 3. research results of physician questionnaires about the clinical utility, accuracy, ease of use, and completeness of GTRx.
